# Supplementary material for: Effects of narrow linear clearings on movement and habitat use in a boreal forest mammal community during winter
Source: PeerJ. 2019 Feb 25;7:e6504. doi: 10.7717/peerj.6504 (PMC6394345; doi:10.7717/peerj.6504)
Supplement: Supplemental Information 2 — Example calculations for deer. [file peerj-07-6504-s002.docx]

**Calculations supplement: Example calculations for deer**

This document provides supplementary instructions to those currently included in the Methods section of the text. It uses an example for one taxon (deer) to describe the process used to calculate the results shown in Figures 2 and 3 and Supplementary Tables 1, 2 and 3 from the raw data file. The process described can be repeated for any of the other taxa or functional groups included in the paper.

1. Using the raw data file, sum records for deer (de) by SL_FO (i.e. transect type=forest or seismic line), TYPE_CD (i.e. crossing (C), approach (A), linear travel distance (D)) by Site_no (i.e. study site). Note that site numbers 9 and 13 were dropped from this study early in the field program due to industrial development activities on these sites. Results (Table 1, below) are interpreted as follows:

On site one (line 1), over nine surveys (3 years) we recorded:

- - 19 deer tracks which approached the forest transect but which did not completely cross. 41 such tracks were recorded over the same period on the paired seismic line.
  - 336 deer tracks which completely crossed the forest transect and 260 which completely crossed the paired seismic line transect.
  - 586 m of deer track, inside the forest transect, which continued in a straight, or diagonal linear path, along the transect, where each individual track was at least 10 m in length (measured parallel to the transect edge). We recorded 3,756 m of such track on the paired seismic line transect.

**Table 1:** Sum of deer tracks recorded on 14 sites, over all forest and seismic line transect (1.0 km each) surveys (9 over 3 years). Units for approaches (A) and crossings (C) are tracks per 9 km and linear travel distance (D) meters of track per 9 km surveyed.

|  | Forest transects | | |  | Seismic line transects | | |
| --- | --- | --- | --- | --- | --- | --- | --- |
| Site_no | A | C | D |  | A | C | D |
| 1 | 19 | 336 | 586 |  | 41 | 260 | 3,756 |
| 2 | 13 | 263 | 220 |  | 3 | 194 | 1,301 |
| 3 | 8 | 374 | 481 |  | 13 | 193 | 882 |
| 4 | 11 | 186 | 327 |  | 9 | 120 | 359 |
| 5 | 15 | 603 | 922 |  | 5 | 282 | 1,229 |
| 6 | 13 | 297 | 157 |  | 9 | 154 | 590 |
| 7 | 3 | 164 | 447 |  | 9 | 107 | 1,802 |
| 8 | 6 | 208 | 193 |  | 5 | 104 | 2,308 |
| 10 | 9 | 129 | 412 |  | 4 | 62 | 399 |
| 11 | 10 | 212 | 148 |  | 4 | 112 | 2,167 |
| 12 | 12 | 425 | 197 |  | 29 | 331 | 3,771 |
| 14 | 5 | 140 | 133 |  | 2 | 47 | 948 |
| 15 | 10 | 436 | 390 |  | 9 | 229 | 2,739 |
| 16 | 3 | 128 | 26 |  | 2 | 54 | 645 |

1. Divide all values by 9 km (9 repeat one km surveys per transect).

**Table 2:** Average deer tracks recorded on 14 sites, each containing one forest and one seismic line transect (1.0 km each), over 9 repeat surveys (3 years). Units for approaches (A) and crossings (C) are tracks per 1 km and linear travel distance (D) is meters of track per 1 km surveyed.

|  | Forest transects | | |  | Seismic line transects | | |
| --- | --- | --- | --- | --- | --- | --- | --- |
| Site_no | A | C | D |  | A | C | D |
| 1 | 2.1 | 37.3 | 65.1 |  | 4.6 | 28.9 | 417.3 |
| 2 | 1.4 | 29.2 | 24.4 |  | 0.3 | 21.6 | 144.6 |
| 3 | 0.9 | 41.6 | 53.4 |  | 1.4 | 21.4 | 98.0 |
| 4 | 1.2 | 20.7 | 36.3 |  | 1.0 | 13.3 | 39.9 |
| 5 | 1.7 | 67.0 | 102.4 |  | 0.6 | 31.3 | 136.6 |
| 6 | 1.4 | 33.0 | 17.4 |  | 1.0 | 17.1 | 65.6 |
| 7 | 0.3 | 18.2 | 49.7 |  | 1.0 | 11.9 | 200.2 |
| 8 | 0.7 | 23.1 | 21.4 |  | 0.6 | 11.6 | 256.4 |
| 10 | 1.0 | 14.3 | 45.8 |  | 0.4 | 6.9 | 44.3 |
| 11 | 1.1 | 23.6 | 16.4 |  | 0.4 | 12.4 | 240.8 |
| 12 | 1.3 | 47.2 | 21.9 |  | 3.2 | 36.8 | 419.0 |
| 14 | 0.6 | 15.6 | 14.8 |  | 0.2 | 5.2 | 105.3 |
| 15 | 1.1 | 48.4 | 43.3 |  | 1.0 | 25.4 | 304.3 |
| 16 | 0.3 | 14.2 | 2.9 |  | 0.2 | 6.0 | 71.7 |

1. Calculate crossing propensity (100C/(C+A)), linear travel propensity (100D/(D+8C)) and habitat usage intensity (D+8C) using Table 2 above, for each site.

**Table 3:** Measures of individuals’ responses to the presence of each transect for deer. Units for approaches (A) and crossings (C) are % and habitat usage intensity units are meters per kilometer surveyed.

|  | Crossing propensity^a^ | |  | Linear travel propensity^b^ | |  | Habitat usage intensity^c^ | |
| --- | --- | --- | --- | --- | --- | --- | --- | --- |
| Site_no | Forest | Seismic line |  | Forest | Seismic line |  | Forest | Seismic line |
| 1 | 94.6 | 86.4 |  | 17.9 | 64.4 |  | 363.8 | 648.4 |
| 2 | 95.3 | 98.5 |  | 9.5 | 45.6 |  | 258.2 | 317.0 |
| 3 | 97.9 | 93.7 |  | 13.8 | 36.4 |  | 385.9 | 269.6 |
| 4 | 94.4 | 93.0 |  | 18.0 | 27.2 |  | 201.7 | 146.6 |
| 5 | 97.6 | 98.3 |  | 16.0 | 35.3 |  | 638.4 | 387.2 |
| 6 | 95.8 | 94.5 |  | 6.2 | 32.4 |  | 281.4 | 202.4 |
| 7 | 98.2 | 92.2 |  | 25.4 | 67.8 |  | 195.4 | 295.3 |
| 8 | 97.2 | 95.4 |  | 10.4 | 73.5 |  | 206.3 | 348.9 |
| 10 | 93.5 | 93.9 |  | 28.5 | 44.6 |  | 160.4 | 99.4 |
| 11 | 95.5 | 96.6 |  | 8.0 | 70.7 |  | 204.9 | 340.3 |
| 12 | 97.3 | 91.9 |  | 5.5 | 58.7 |  | 399.7 | 713.2 |
| 14 | 96.6 | 95.9 |  | 10.6 | 71.6 |  | 139.2 | 147.1 |
| 15 | 97.8 | 96.2 |  | 10.1 | 59.9 |  | 430.9 | 507.9 |
| 16 | 97.7 | 96.4 |  | 2.5 | 59.9 |  | 116.7 | 119.7 |
| Mean | **96.4** | **94.5** |  | **13.0** | **53.4** |  | **284.5** | **324.5** |
| S.E. | **0.4** | **0.8** |  | **2.0** | **4.3** |  | **38.5** | **50.8** |

1. *Mean and S.E. match Supplementary Table 1 for the “deer” row and the values for “deer” in figure 2a*
2. *Mean and S.E. match Supplementary Table 2 for the “deer” row and the values for “deer” in figure 2b*
3. *Mean and S.E. match Supplementary Table 3 for the “deer” row and the values for “deer” in figure 2c*
4. Differences between seismic line and forest transects in crossing propensity, linear travel propensity and habitat usage intensity were tested using paired t-tests (n=14 transect pairs).
