# Supplementary material for: Effects of narrow linear clearings on movement and habitat use in a boreal forest mammal community during winter
Source: PeerJ. 2019 Feb 25;7:e6504. doi: 10.7717/peerj.6504 (PMC6394345; doi:10.7717/peerj.6504)
Supplement: Supplemental Information 3 — Mean crossing propensity (%, standard error) for forest and seismic line transects (9 surveys) with t-test p-values (N = 14 paired sites). [file peerj-07-6504-s003.docx]

Supplementary Table 1: Mean crossing propensity (%, standard error) for forest and seismic line transects (9 surveys) with t-test *p*-values (n=14 paired sites; where normality assumption violated Wilcoxon *p*-value also shown), mean difference, 95% confidence interval and Cohen’s effect size for correlated measurements (*d_z_*). Bold indicates significant Benjamini-Hochberg result, but *p*-values here are unadjusted for false discovery (Supplementary Table 7 contains adjusted *p*-values).

|  |  | Forest | | Seismic line | |  |  |  | Confidence Interval^1^ | |  |
| --- | --- | --- | --- | --- | --- | --- | --- | --- | --- | --- | --- |
| Taxonomic group | | % | SE | % | SE | *P* | *Wilc p* | Mean diff.^1^ | Lower | Upper | *d_z_* |
|  | Cougar | 84.7 | 9.6 | 78.6 | 11.4 | 0.640 |  | 6.1 | -21.5 | 33.7 | 0.13 |
|  | Gray wolf | 48.2 | 13.4 | 48.7 | 13.6 | 0.975 |  | -0.5 | -32.8 | 31.8 | -0.01 |
|  | Coyote | 89.2 | 7.0 | 80.1 | 9.8 | 0.496 |  | 9.1 | -19.0 | 37.2 | 0.19 |
|  | Lynx | 82.8 | 9.5 | 69.6 | 12.3 | 0.384 |  | 13.1 | -18.4 | 44.6 | 0.24 |
|  | Marten | 85.7 | 6.7 | 84.8 | 6.7 | 0.703 |  | 1.0 | -4.4 | 6.3 | 0.10 |
|  | Weasel | 78.6 | 9.4 | 70.0 | 8.8 | 0.537 |  | 8.6 | -20.7 | 38.0 | 0.17 |
|  | Moose & elk | 95.2 | 2.1 | 93.1 | 2.0 | 0.516 |  | 2.1 | -4.6 | 8.8 | 0.18 |
|  | Deer | 96.4 | 0.4 | 94.5 | 0.8 | 0.040 |  | 1.9 | 0.1 | 3.7 | 0.61 |
|  | Hare | **96.5** | 0.8 | 87.5 | 1.8 | **<0.001** | **0.003** | 9.0 | 5.4 | 12.6 | **1.45** |
|  | Red squirrel | 95.0 | 1.0 | 95.4 | 0.7 | 0.753 |  | -0.4 | -3.0 | 2.2 | -0.09 |
|  | Mouse | 64.8 | 3.5 | 75.1 | 5.7 | 0.115 |  | -10.3 | -23.6 | 2.9 | -0.45 |
|  | Vole | 71.2 | 2.7 | 73.4 | 4.4 | 0.583 |  | -2.3 | -11.0 | 6.4 | -0.15 |
|  | Shrew | 59.6 | 4.3 | 70.6 | 6.6 | 0.104 |  | -11.0 | -24.7 | 2.6 | -0.47 |
| Body size-diet group | |  |  |  |  |  |  |  |  |  |  |
|  | Large predators | 94.8 | 1.4 | 96.8 | 1.6 | 0.360 |  | -2.0 | -6.6 | 2.6 | -0.25 |
|  | Mid-sized predators | **92.7** | 1.0 | 86.5 | 1.6 | **0.007** | **0.007** | 6.2 | 2.1 | 10.4 | **0.86** |
|  | Large herbivores | **96.3** | 0.5 | 93.9 | 0.8 | **0.012** | **0.017** | 2.4 | 0.6 | 4.2 | **0.78** |
|  | Mid-sized herbivores | 95.5 | 0.7 | 92.3 | 1.2 | 0.037 | 0.030 | 3.2 | 0.2 | 6.3 | 0.62 |
|  | Small mammals | 67.0 | 2.5 | 74.4 | 4.0 | 0.077 |  | -7.4 | -15.7 | 0.9 | -0.51 |
| All species | | 92.3 | 0.9 | 89.4 | 1.2 | 0.074 |  | 3.0 | -0.3 | 6.2 | 0.52 |

1. Negative values indicate crossing propensity for forest transects was less than crossing propensity for seismic line transects.
