# Supplementary material for: Effects of narrow linear clearings on movement and habitat use in a boreal forest mammal community during winter
Source: PeerJ. 2019 Feb 25;7:e6504. doi: 10.7717/peerj.6504 (PMC6394345; doi:10.7717/peerj.6504)
Supplement: Supplemental Information 4 — Mean linear travel propensity (%, standard error) for forest and seismic line transects (9 surveys) with t-test p-values (N = 14 paired sites). [file peerj-07-6504-s004.docx]

Supplementary Table 2: Mean linear travel propensity (%, standard error) for forest and seismic line transects (9 surveys) with t-test *p*-values (n=14 paired sites; where normality assumption violated Wilcoxon *p*-value also shown), mean difference, 95% confidence interval and Cohen’s effect size for correlated measurements (*d_z_*). Bold indicates significant Benjamini-Hochberg result, but *p*-values here are unadjusted for false detection (Supplementary Table 8 contains adjusted *p*-values).

|  |  | Forest | | Seismic line | |  |  |  | Confidence Interval | |  |
| --- | --- | --- | --- | --- | --- | --- | --- | --- | --- | --- | --- |
| Taxonomic group | | % | SE | % | SE | *p* | *Wilc p* | Mean diff. | Lower | Upper | *d_z_* |
|  | Cougar | 0.3 | 0.3 | **46.9** | 13.0 | ***0.003*** | ***0.022*** | -46.5 | -74.5 | -18.6 | **-0.96** |
|  | Gray wolf | 5.5 | 3.7 | **57.6** | 12.7 | ***<0.001*** | ***0.009*** | -52.2 | -77.8 | -26.6 | **-1.18** |
|  | Coyote | 19.5 | 7.5 | **93.3** | 2.5 | ***<0.001*** | ***0.001*** | -73.8 | -92.3 | -55.2 | **-2.29** |
|  | Lynx | 2.2 | 1.5 | 23.2 | 10.4 | *0.065* |  | -21.0 | -43.5 | 1.5 | -0.54 |
|  | Marten | 3.6 | 1.1 | 8.1 | 3.5 | *0.226* |  | -4.5 | -12.1 | 3.1 | -0.34 |
|  | Weasel | 1.3 | 1.3 | 15.6 | 8.6 | *0.100* |  | -14.4 | -31.9 | 3.2 | -0.47 |
|  | Moose & elk | 12.0 | 4.8 | **39.1** | 7.3 | ***0.001*** |  | -27.1 | -41.5 | -12.7 | **-1.09** |
|  | Deer | 13.0 | 2.0 | **53.4** | 4.3 | ***<0.001*** | ***<0.001*** | -40.4 | -51.2 | -29.6 | **-2.15** |
|  | Hare | 7.3 | 3.2 | 15.4 | 5.0 | *0.165* |  | -8.1 | -20.0 | 3.8 | -0.39 |
|  | Red squirrel | 0.1 | 0.0 | 1.3 | 0.6 | *0.052* |  | -1.2 | -2.4 | 0.0 | -0.57 |
|  | Mouse | 0.4 | 0.4 | 4.9 | 4.5 | *0.295* |  | -4.4 | -13.2 | 4.3 | -0.29 |
|  | Vole | 0.2 | 0.1 | 1.8 | 1.8 | *0.358* |  | -1.6 | -5.2 | 2.0 | -0.25 |
|  | Shrew | 0.5 | 0.4 | 0.0 | 0.0 | *0.168* |  | 0.5 | -0.2 | 1.3 | 0.39 |
| Body size-diet group | |  |  |  |  |  |  |  |  |  |  |
|  | Large predators | 19.0 | 5.8 | **92.3** | 2.1 | ***<0.001*** | ***<0.001*** | -73.3 | -86.4 | -60.1 | **-3.22** |
|  | Mid-sized predators | 3.4 | 1.1 | **14.8** | 4.0 | ***0.013*** | ***0.013*** | -11.4 | -19.9 | -2.9 | **-0.77** |
|  | Large herbivores | 13.6 | 2.1 | **54.2** | 3.6 | ***<0.001*** | ***<0.001*** | -40.7 | -49.7 | -31.7 | **-2.60** |
|  | Mid-sized herbivores | 2.6 | 1.5 | 5.1 | 1.6 | *0.203* |  | -2.6 | -6.7 | 1.6 | -0.36 |
|  | Small mammals | 0.4 | 0.3 | 3.8 | 3.6 | *0.321* |  | -3.4 | -10.7 | 3.8 | -0.28 |
| Total all species | | 5.9 | 1.1 | **41.7** | 3.7 | ***<0.001*** | ***<0.001*** | -35.8 | -43.8 | -27.8 | **-2.59** |

1. Negative values indicate linear travel propensity for forest transects was less than travel propensity for seismic line transects.
