# Supplementary material for: Effects of narrow linear clearings on movement and habitat use in a boreal forest mammal community during winter
Source: PeerJ. 2019 Feb 25;7:e6504. doi: 10.7717/peerj.6504 (PMC6394345; doi:10.7717/peerj.6504)
Supplement: Supplemental Information 5 — Mean habitat usage intensity (m/km; standard error) for forest and seismic line transects (9 surveys) with t-test p-values (N = 14 paired sites). [file peerj-07-6504-s005.docx]

Supplementary Table 3: Mean habitat usage intensity (m/km; standard error and confidence interval) for forest and seismic line transects (9 surveys) and where assumptions were violated (normality, equal variance, no outliers), the transformation applied to conduct paired t-test analyses (n=14 paired sites). Also shown are mean differences and 95% confidence interval for the transformed variables. Bold indicates a significant Benjamini-Hochberg result, but *p*-values here are unadjusted for false detection (Supplementary Table 9 contains adjusted *p*-values). Cohen’s effect size for correlated measurements (*d_z_*) is provided.

|  |  | Forest | | Seismic line | |  |  |  |  | Transformed results | |  |  |
| --- | --- | --- | --- | --- | --- | --- | --- | --- | --- | --- | --- | --- | --- |
|  |  | m | SE | m | SE | Mean diff. |  | *Trans.* | Mean diff. | Confidence Interval | | *P* | d*_z_* |
| Taxonomic group | |  |  |  |  |  |  |  |  | Lower | Upper |  |  |
|  | Cougar | 3.3 | 1.0 | 25.9 | 12.6 | -22.5 |  | 3rd | -0.8 | -1.58 | 0.04 | 0.060 | -0.550 |
|  | Gray wolf | 4.0 | 1.9 | **78.1** | 28.5 | -74.0 |  | log | -1.8 | -2.90 | -0.66 | **0.005** | **-0.915** |
|  | Coyote | 16.0 | 4.5 | **259.6** | 75.7 | -243.6 |  | 3rd | -3.5 | -5.02 | -2.05 | **<0.001** | **-1.373** |
|  | Lynx | 4.2 | 1.5 | 11.1 | 5.7 | -6.9 |  | 3rd | -0.1 | -0.78 | 0.54 | 0.700 | -0.105 |
|  | Marten | **71.1** | 15.4 | 40.9 | 9.8 | 30.2 |  | na | -8.4 | 13.40 | 47.02 | **0.002** | **1.037** |
|  | Weasel | 8.6 | 2.9 | 17.0 | 7.8 | -8.4 |  | 2nd | -0.8 | -2.08 | 0.42 | 0.177 | -0.382 |
|  | Moose & elk | 19.1 | 5.4 | **36.4** | 7.8 | -17.3 |  | 2nd | -1.7 | -3.03 | -0.30 | **0.021** | **-0.703** |
|  | Deer | 284.5 | 38.5 | 324.5 | 50.8 | -40.0 |  | na | -40.0 | -128.40 | 48.39 | 0.346 | -0.261 |
|  | Hare | 318.8 | 127.1 | 177.3 | 61.6 | 141.4 |  | 4th | 0.3 | -0.16 | 0.84 | 0.162 | 0.396 |
|  | Red squirrel | 549.4 | 150.1 | 325.9 | 54.6 | 223.5 |  | 2nd | 3.4 | -1.69 | 8.45 | 0.174 | 0.385 |
|  | Mouse | 22.9 | 3.5 | 32.0 | 12.0 | -9.1 |  | 4th | 0.0 | -0.34 | 0.30 | 0.910 | -0.031 |
|  | Vole | 39.5 | 7.0 | 40.9 | 6.6 | -1.4 |  | 4th | 0.0 | -0.13 | 0.07 | 0.494 | -0.188 |
|  | Shrew | 30.2 | 6.5 | 24.1 | 6.2 | 6.1 |  | 3rd | 0.2 | -0.05 | 0.53 | 0.097 | 0.478 |
| Body size-diet group | |  |  |  |  |  |  |  |  |  |  |  |  |
|  | Large predator | 27.6 | 4.9 | **380.0** | 81.0 | -352.4 |  | 2nd | -13.0 | -18.07 | -7.99 | **<0.001** | **-1.494** |
|  | Mid-sized predator | **81.2** | 15.4 | 60.2 | 13.1 | 21.0 |  | na | 21.0 | 0.98 | 41.04 | **0.041** | **0.606** |
|  | Large herbivore | 303.6 | 39.0 | 361.0 | 48.0 | -57.4 |  | 2nd | -1.5 | -3.48 | 0.55 | 0.140 | -0.420 |
|  | Mid-sized herbivore | **868.2** | 187.8 | 503.2 | 69.3 | 365.0 |  | 2nd | 5.2 | 0.43 | 9.92 | **0.035** | **0.630** |
|  | Small mammal | 92.6 | 11.7 | 97.0 | 17.0 | -4.4 |  | 2nd | 0.0 | -0.89 | 0.81 | 0.918 | -0.028 |
| All species | | 1,373.1 | 175.0 | 1,401.3 | 118.0 | -28.2 |  | 2nd | -1.0 | -6.30 | 4.30 | 0.690 | -0.109 |

1. In total, 126 km were surveyed for each transect type. Multiple individuals were sometimes recorded simultaneously and multiple species are combined for body size-diet groups.
2. Transformations were applied to data sets prior to conducting paired *t*-tests. 2^nd^ indicates square root transform, 3^rd^ is cubed root, etc., na indicates that a transformation as not applied, log applied as log(x+1).
3. Negative values indicate that habitat usage intensity on forest transects was less than habitat usage on seismic line transects.
