# Supplementary material for: Effects of narrow linear clearings on movement and habitat use in a boreal forest mammal community during winter
Source: PeerJ. 2019 Feb 25;7:e6504. doi: 10.7717/peerj.6504 (PMC6394345; doi:10.7717/peerj.6504)
Supplement: Supplemental Information 6 — Mean habitat usage intensity (m/sub-transect; standard error) for 100 m sub-transects which were located at various distances from the nearest seismic line (0–50 m; 51–150 m and 151–570 m) and means of log transformations of these values which were used to conduct repeated measures analysis of variance (ANOVA) where sites (n = 14) were subjects which were measured repeatedly with track surveys of sub-transects (140 sub-transects). Also reported are repeated measures ANOVA p-values, Greenhouse-Geisser corrected p-values where sphericity was violated (Gg p), results of Shapiro-Wilk tests of residuals for normality (Sh-Wi p), generalized eta squared (ηG2) and partial eta squared (ηp2) which indicate effect size associated with proximity to seismic lines. [file peerj-07-6504-s006.docx]

|  |  | Meters of track per 100 m | | | | | |  | Log meters of track per 100 m | | | | | | | | | | |
| --- | --- | --- | --- | --- | --- | --- | --- | --- | --- | --- | --- | --- | --- | --- | --- | --- | --- | --- | --- |
|  |  | 0-50 | | 51-150 | | 151-570 | |  | 0-50 | | 51-150 | | 151-570 | |  |  |  |  |  |
| Taxonomic group | | Mean | SE | Mean | SE | Mean | SE |  | Mean | SE | Mean | SE | Mean | SE | *p* | Gg *p* | *Sh-Wi p* | *η_G_²* | *η_p_²* |
|  | Cougar | 0.2 | 0.1 | 0.3 | 0.1 | 0.4 | 0.1 |  | 0.14 | 0.06 | 0.18 | 0.05 | 0.24 | 0.07 | *0.401* |  | *<0.001* | *0.036* | *0.036* |
|  | Gray wolf | 0.5 | 0.3 | 0.4 | 0.2 | 0.4 | 0.2 |  | 0.19 | 0.11 | 0.20 | 0.09 | 0.19 | 0.10 | *0.996* | *0.966* | *<0.001* | *<0.001* | *<0.001* |
|  | Coyote | 1.7 | 0.8 | 1.2 | 0.3 | 1.4 | 0.6 |  | 0.57 | 0.20 | 0.57 | 0.14 | 0.49 | 0.13 | *0.878* | *0.806* | *<0.001* | *0.003* | *0.010* |
|  | Lynx | 0.3 | 0.1 | 0.7 | 0.3 | 0.4 | 0.2 |  | 0.16 | 0.08 | 0.29 | 0.12 | 0.19 | 0.09 | *0.544* |  | *<0.001* | *0.026* | *0.046* |
|  | Marten | 6.5 | 1.4 | 8.2 | 2.1 | 6.4 | 1.7 |  | 1.60 | 0.26 | 1.76 | 0.27 | 1.51 | 0.27 | *0.382* |  | *0.036* | *0.012* | *0.071* |
|  | Weasel | 0.5 | 0.3 | 1.1 | 0.4 | 0.9 | 0.4 |  | 0.26 | 0.12 | 0.42 | 0.14 | 0.38 | 0.13 | *0.329* | *0.313* | *<0.001* | *0.022* | *0.082* |
|  | Moose/elk | 4.1 | 1.4 | 1.8 | 0.6 | 1.4 | 0.5 |  | 1.21 | 0.23 | 0.70 | 0.18 | 0.60 | 0.15 | ***<0.001*** |  | ***0.007*** | ***0.135*** | ***0.451*** |
|  | Deer | 32.4 | 7.0 | 25.7 | 4.4 | 30.2 | 3.8 |  | 3.23 | 0.19 | 3.27 | 0.23 | 2.97 | 0.13 | *0.120* |  | *0.045* | *0.036* | *0.150* |
|  | Hare | 34.0 | 13.9 | 41.1 | 16.5 | 33.9 | 14.7 |  | 2.11 | 0.50 | 2.01 | 0.51 | 2.11 | 0.48 | *0.926* |  | *<0.001* | *0.001* | *0.006* |
|  | Red squirrel | 52.7 | 16.6 | 41.7 | 11.1 | 58.5 | 17.6 |  | 3.16 | 0.40 | 3.17 | 0.31 | 3.35 | 0.36 | *0.740* |  | *0.132* | *0.005* | *0.023* |
|  | Vole | 4.2 | 1.3 | 4.3 | 0.7 | 4.0 | 0.9 |  | 1.24 | 0.21 | 1.45 | 0.10 | 1.22 | 0.10 | *0.374* |  | *0.209* | *0.026* | *0.073* |
|  | Mouse | 2.1 | 0.4 | 3.3 | 1.0 | 1.8 | 0.4 |  | 0.94 | 0.16 | 1.03 | 0.20 | 0.71 | 0.12 | *0.249* |  | *0.188* | *0.048* | *0.101* |
|  | Shrew | 2.0 | 0.6 | 3.6 | 0.8 | 3.7 | 1.2 |  | 0.74 | 0.20 | 1.10 | 0.18 | 1.07 | 0.23 | *0.091* | *0.114* | *0.059* | *0.048* | *0.168* |
| Body size-diet group | |  |  |  |  |  |  |  |  |  |  |  |  |  |  |  |  |  |  |
|  | Large predator | 2.7 | 0.8 | 2.6 | 0.4 | 2.6 | 0.6 |  | 0.95 | 0.19 | 1.01 | 0.14 | 0.98 | 0.13 | *0.883* |  | *0.570* | *0.002* | *0.010* |
|  | Mid-sized predator | 7.1 | 1.4 | 9.5 | 2.2 | 7.5 | 1.6 |  | 1.78 | 0.23 | 1.99 | 0.23 | 1.75 | 0.23 | *0.289* |  | *0.086* | *0.015* | *0.091* |
|  | Large herbivore | 36.5 | 7.5 | 27.6 | 4.4 | 31.7 | 3.8 |  | 3.37 | 0.16 | 3.14 | 0.16 | 3.33 | 0.13 | *0.113* |  | *0.984* | *0.034* | *0.154* |
|  | Mid-sized herbivore | 86.7 | 19.6 | 82.8 | 19.4 | 92.5 | 20.8 |  | 3.83 | 0.36 | 3.75 | 0.32 | 3.97 | 0.36 | *0.683* |  | *0.009* | *0.005* | *0.029* |
|  | Small mammal | 8.3 | 1.8 | 11.1 | 1.3 | 9.6 | 1.9 |  | 1.95 | 0.19 | 2.29 | 0.13 | 2.05 | 0.18 | *0.212* |  | *0.847* | *0.049* | *0.113* |
|  | All taxa | 141.3 | 17.6 | 133.6 | 18.1 | 143.9 | 19.9 |  | 4.79 | 0.14 | 4.73 | 0.16 | 4.80 | 0.16 | *0.688* |  | *0.055* | *0.003* | *0.028* |

Supplementary Table 4: Mean habitat usage intensity (m/sub-transect; standard error) for 100 m sub-transects which were located at various distances from the nearest seismic line (0-50 m; 51-150 m and 151-570 m) and means of log transformations of these values which were used to conduct repeated measures analysis of variance (ANOVA) where sites (n=14) were subjects which were measured repeatedly with track surveys of sub-transects (140 sub-transects). Also reported are repeated measures ANOVA *p*-values, Greenhouse-Geisser corrected *p-*values where sphericity was violated (Gg *p*), results of Shapiro-Wilk tests of residuals for normality (Sh-Wi *p*), generalized eta squared (η_G_²) and partial eta squared (*η_p_²*) which indicate effect size associated with proximity to seismic lines.
