# Supplementary material for: Effects of narrow linear clearings on movement and habitat use in a boreal forest mammal community during winter
Source: PeerJ. 2019 Feb 25;7:e6504. doi: 10.7717/peerj.6504 (PMC6394345; doi:10.7717/peerj.6504)
Supplement: Supplemental Information 7 — Crossing propensity results of post-hoc power (1-β) analysis using observed variance, for paired t-tests (n = 14 paired transects) to detect a mean difference between seismic line and forest transects (α = 0.05), of 20%, 50% or 80%, if one existed. Results shown where an effect was not detected. [file peerj-07-6504-s007.docx]

Supplementary Table 5: Crossing propensity results of post-hoc power (1-β) analysis using observed variance, for paired t-tests (n=14 paired transects) to detect a mean difference between seismic line and forest transects (α=0.05), of 20%, 50% or 80%, if one existed. Results shown where an effect was not detected.

|  |  | Mean crossing propensity difference | | |
| --- | --- | --- | --- | --- |
| Taxonomic group | | 20% | 50% | 80% |
|  | Cougar | 0.303 | 0.949 | 0.999 |
|  | Gray wolf | 0.386 | 0.870 | 0.998 |
|  | Coyote | 0.389 | 0.987 | 0.999 |
|  | Lynx | 0.249 | 0.891 | 0.999 |
|  | Marten | >0.999 | >0.999 | >0.999 |
|  | Weasel | 0.334 | 0.968 | 0.999 |
|  | Moose & elk | 0.999 | >0.999 | >0.999 |
|  | Deer | - | - | - |
|  | Hare | - | - | - |
|  | Red squirrel | >0.999 | >0.999 | >0.999 |
|  | Mouse | 0.854 | >0.999 | >0.999 |
|  | Vole | 0.996 | >0.999 | >0.999 |
|  | Shrew | 0.826 | 0.999 | >0.999 |
| Body size-diet group | |  |  |  |
|  | Large predators | >0.999 | >0.999 | >0.999 |
|  | Mid-sized predators | - | - | - |
|  | Large herbivores | - | - | - |
|  | Mid-sized herbivores | - | - | - |
|  | Small mammals | 0.998 | >0.999 | >0.999 |
| All species | | >0.999 | >0.999 | >0.999 |
