# Supplementary material for: Effects of narrow linear clearings on movement and habitat use in a boreal forest mammal community during winter
Source: PeerJ. 2019 Feb 25;7:e6504. doi: 10.7717/peerj.6504 (PMC6394345; doi:10.7717/peerj.6504)
Supplement: Supplemental Information 8 — Travel propensity results of post-hoc power (1-β) analysis using observed variance, for paired t-tests (n = 14 paired transects) to detect a mean difference between seismic line and forest transects (α = 0.05), of 20%, 50% or 80%, if one existed. Results shown where an effect was not detected. [file peerj-07-6504-s008.docx]

Supplementary Table 6: Travel propensity results of post-hoc power (1-β) analysis using observed variance, for paired t-tests (n=14 paired transects) to detect a mean difference between seismic line and forest transects (α=0.05), of 20%, 50% or 80%, if one existed. Results shown where an effect was not detected.

|  |  | Mean travel propensity difference | | |
| --- | --- | --- | --- | --- |
| Taxonomic group | | 20% | 50% | 80% |
|  | Cougar | - | - | - |
|  | Gray wolf | - | - | - |
|  | Coyote | - | - | - |
|  | Lynx | 0.429 | 0.993 | 0.999 |
|  | Marten | >0.999 | >0.999 | >0.999 |
|  | Weasel | - | - | - |
|  | Moose & elk | - | - | - |
|  | Deer | - | - | - |
|  | Hare | 0.910 | >0.999 | >0.999 |
|  | Red squirrel | >0.999 | >0.999 | >0.999 |
|  | Mouse | 0.994 | >0.999 | >0.999 |
|  | Vole | >0.999 | >0.999 | >0.999 |
|  | Shrew | >0.999 | >0.999 | >0.999 |
| Body size-diet group | |  |  |  |
|  | Large predators | - | - | - |
|  | Mid-sized predators | - | - | - |
|  | Large herbivores | - | - | - |
|  | Mid-sized herbivores | >0.999 | >0.999 | >0.999 |
|  | Small mammals | >0.999 | >0.999 | >0.999 |
| All species | | - | - | - |
