# Supplementary material for: Effects of narrow linear clearings on movement and habitat use in a boreal forest mammal community during winter
Source: PeerJ. 2019 Feb 25;7:e6504. doi: 10.7717/peerj.6504 (PMC6394345; doi:10.7717/peerj.6504)
Supplement: Supplemental Information 9 — Habitat usage intensity results of post-hoc power (1-β) analysis using observed variance, for paired t-tests (n = 14 paired transects) to detect a mean difference between seismic line and forest transects (α = 0.05), of 20%, 50% or 80%, if one existed. Results shown where an effect was not detected. [file peerj-07-6504-s009.docx]

Supplementary Table 7: Habitat usage intensity results of post-hoc power (1-β) analysis using observed variance, for paired t-tests (n=14 paired transects) to detect a mean difference between seismic line and forest transects (α=0.05), of 20%, 50% or 80%, if one existed. Results shown where an effect was not detected.

|  |  | Mean usage intensity difference | | |
| --- | --- | --- | --- | --- |
| Taxonomic group | | 20% | 50% | 80% |
|  | Cougar | 0.066 | 0.164 | 0.350 |
|  | Gray wolf | - | - | - |
|  | Coyote | - | - | - |
|  | Lynx | 0.063 | 0.173 | 0.329 |
|  | Marten | - | - | - |
|  | Weasel | 0.073 | 0.269 | 0.549 |
|  | Moose & elk | - | - | - |
|  | Deer | 0.313 | 0.957 | 0.999 |
|  | Hare | 0.103 | 0.382 | 0.756 |
|  | Red squirrel | 0.143 | 0.565 | 0.940 |
|  | Mouse | 0.081 | 0.283 | 0.612 |
|  | Vole | 0.717 | >0.999 | >0.999 |
|  | Shrew | 0.315 | 0.957 | >0.999 |
| Body size-diet group | |  |  |  |
|  | Large predators | - | - | - |
|  | Mid-sized predators | - | - | - |
|  | Large herbivores | 0.397 | 0.988 | >0.999 |
|  | Mid-sized herbivores | - | - | - |
|  | Small mammals | 0.450 | 0.996 | >0.999 |
| All species | | 0.314 | 0.956 | >0.999 |
