# Supplementary material for: Effects of narrow linear clearings on movement and habitat use in a boreal forest mammal community during winter
Source: PeerJ. 2019 Feb 25;7:e6504. doi: 10.7717/peerj.6504 (PMC6394345; doi:10.7717/peerj.6504)
Supplement: Supplemental Information 10 — Crossing propensity results of Benjamini-Hochberg procedure to correct for false discovery over 19 paired t-tests (n = 14 paired sites) for a significant difference between seismic line and forest transects, with a false discovery rate (FDR) of 0.15. Mammals sorted by unadjusted p-value, bold indicates p < 0.05 and the Benjamini-Hochberg procedure declared a significant result. [file peerj-07-6504-s010.docx]

Supplementary Table 8: Crossing propensity results of Benjamini-Hochberg procedure to correct for false discovery over 19 paired t-tests (n=14 paired sites) for a significant difference between seismic line and forest transects, with a false discovery rate (FDR) of 0.15. Mammals sorted by unadjusted *p*-value, bold indicates *p*<0.05 and the Benjamini-Hochberg procedure declared a significant result.

| Taxonomic group | Unadjusted *p*-value | FDR-derived significance threshold^1^ | FDR-adjusted *p*-values (*q*-values)^2^ |
| --- | --- | --- | --- |
| Hare | **<0.001** | 0.008 | 0.002 |
| Mid-sized predators | **0.007** | 0.016 | 0.063 |
| Large herbivores | **0.012** | 0.024 | 0.074 |
| Mid-sized herbivores | 0.037 | 0.032 | 0.152 |
| Deer | 0.040 | 0.039 | 0.152 |
| All species | 0.074 | 0.047 | 0.208 |
| Small mammals | 0.077 | 0.055 | 0.208 |
| Shrew | 0.104 | 0.063 | 0.242 |
| Mouse | 0.115 | 0.071 | 0.242 |
| Large predators | 0.360 | 0.079 | 0.664 |
| Lynx | 0.384 | 0.087 | 0.664 |
| Coyote | 0.496 | 0.095 | 0.729 |
| Moose & elk | 0.516 | 0.103 | 0.729 |
| Weasel | 0.537 | 0.111 | 0.729 |
| Vole | 0.583 | 0.118 | 0.738 |
| Cougar | 0.640 | 0.126 | 0.760 |
| Marten | 0.703 | 0.134 | 0.786 |
| Red squirrel | 0.753 | 0.142 | 0.795 |
| Gray wolf | 0.975 | 0.150 | 0.975 |

1. Calculated as *iq/m* where *i* is the *p*-value rank, *q* is the FDR and *m* is number of tests (19). Test declared significant for all tests ranked higher than the test with the largest *p*-value that had *p<iq/m*.
2. Calculated as unadjusted *p*-value times *m/i* or the next adjusted *p*-value in the list, whichever is smaller and interpreted as the probability that the test was truly not significant given that it was declared significant.
