# Supplementary material for: Effects of narrow linear clearings on movement and habitat use in a boreal forest mammal community during winter
Source: PeerJ. 2019 Feb 25;7:e6504. doi: 10.7717/peerj.6504 (PMC6394345; doi:10.7717/peerj.6504)
Supplement: Supplemental Information 13 — Habitat usage intensity results of Benjamini-Hochberg procedure to correct for false discovery over 19 repeated measures ANOVA tests (n = 140 sub-transects) for a significant difference between sub-transects (100 m in length) located varying distances from seismic lines, with a false discovery rate (FDR) of 0.15. Mammals sorted by unadjusted p-value, bold indicates p < 0.05 and the Benjamini-Hochberg procedure declared a significant result. [file peerj-07-6504-s013.docx]

Supplementary Table 11: Habitat usage intensity results of Benjamini-Hochberg procedure to correct for false discovery over 19 repeated measures ANOVA tests (n=140 sub-transects) for a significant difference between sub-transects (100 m in length) located varying distances from seismic lines, with a false discovery rate (FDR) of 0.15. Mammals sorted by unadjusted *p*-value, bold indicates *p*<0.05 and the Benjamini-Hochberg procedure declared a significant result.

| Taxonomic group | Unadjusted *p*-value | FDR-derived significance threshold | FDR-adjusted *p*-values (*q*-values) |
| --- | --- | --- | --- |
| Moose/elk | **<0.001** | 0.008 | 0.008 |
| Large herbivore | 0.113 | 0.016 | 0.570 |
| Shrew | 0.114 | 0.024 | 0.570 |
| Deer | 0.120 | 0.032 | 0.570 |
| Small mammal | 0.212 | 0.039 | 0.692 |
| Mouse | 0.249 | 0.047 | 0.692 |
| Mid-sized predator | 0.289 | 0.055 | 0.692 |
| Weasel | 0.313 | 0.063 | 0.692 |
| Vole | 0.374 | 0.071 | 0.692 |
| Marten | 0.382 | 0.079 | 0.692 |
| Cougar | 0.401 | 0.087 | 0.692 |
| Lynx | 0.544 | 0.095 | 0.862 |
| Mid-sized herbivore | 0.683 | 0.103 | 0.934 |
| All taxa | 0.688 | 0.111 | 0.934 |
| Red squirrel | 0.740 | 0.118 | 0.938 |
| Coyote | 0.806 | 0.126 | 0.957 |
| Large predator | 0.883 | 0.134 | 0.966 |
| Hare | 0.926 | 0.142 | 0.966 |
| Gray wolf | 0.966 | 0.150 | 0.966 |

1. Calculated as *iq/m* where *i* is the *p*-value rank, *q* is the FDR and *m* is number of tests (19). Test declared significant for all tests ranked higher than the test with the largest *p*-value that had *p<iq/m*.
2. Calculated as unadjusted *p*-value times *m/i* or the next adjusted *p*-value in the list, whichever is smaller and interpreted as the probability that the test was truly not significant given that it was declared significant.
